# Molecular Pathogenesis of NASH

**DOI:** 10.3390/ijms17091575

**Published:** 2016-09-20

**Authors:** Alessandra Caligiuri, Alessandra Gentilini, Fabio Marra

**Affiliations:** Dipartimento di Medicina Sperimentale e Clinica, Università degli Studi di Firenze, Firenze 50121, Italy; alessandra.caligiuri@unifi.it (A.C.); alessandra.gentilini@unifi.it (A.G.)

**Keywords:** fibrosis, inflammation, chemokines, genetics, microbiota, pattern-recognition receptors, nuclear receptors, hepatic stellate cells, macrophages

## Abstract

Nonalcoholic steatohepatitis (NASH) is the main cause of chronic liver disease in the Western world and a major health problem, owing to its close association with obesity, diabetes, and the metabolic syndrome. NASH progression results from numerous events originating within the liver, as well as from signals derived from the adipose tissue and the gastrointestinal tract. In a fraction of NASH patients, disease may progress, eventually leading to advanced fibrosis, cirrhosis and hepatocellular carcinoma. Understanding the mechanisms leading to NASH and its evolution to cirrhosis is critical to identifying effective approaches for the treatment of this condition. In this review, we focus on some of the most recent data reported on the pathogenesis of NASH and its fibrogenic progression, highlighting potential targets for treatment or identification of biomarkers of disease progression.

## 1. Introduction

Nonalcoholic fatty liver disease (NAFLD) is an expanding health problem, which varies in prevalence among ethnic groups, occurring with an estimated global prevalence of 25% [1]. NAFLD associates with obesity, insulin resistance or type 2 diabetes and other metabolic abnormalities, such as dyslipidemia and hypertension, collectively termed metabolic syndrome. In high risk populations, the prevalence of NAFLD may be as high as 70%–90% [2,3]. NAFLD covers a spectrum of pathological abnormalities. Although most patients have simple steatosis, around 7%–30% develop nonalcoholic steatohepatitis (NASH), that in at least a third of cases progresses to advanced fibrosis or cirrhosis. The tendency to develop hepatic steatosis differs among ethnic groups, with African-Americans having a lower (24%) and Hispanics a higher (45%) frequency of the disease than Americans of European descent (33%). The causes for these ethnic differences in prevalence of hepatic steatosis and liver injury are not entirely understood.

**Figure 1:**
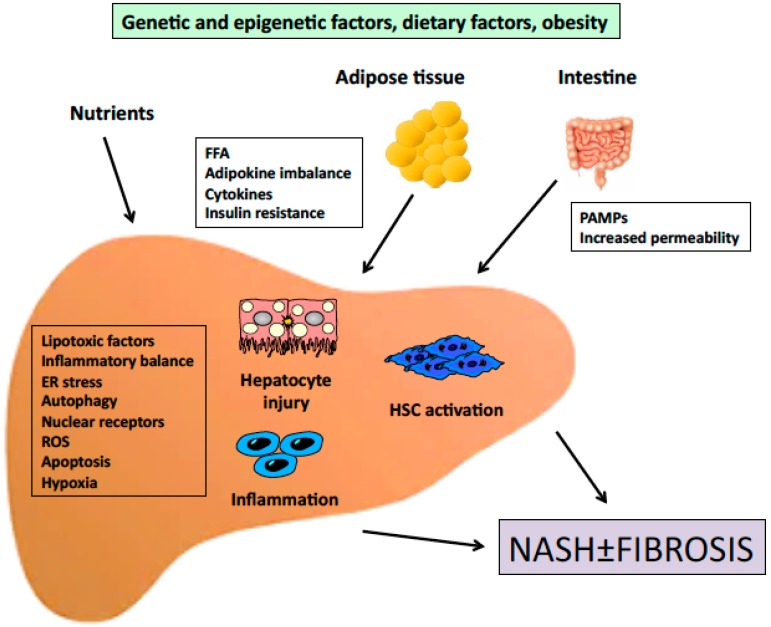
Outline of the pathogenesis of NASH. Signals generated inside the liver as a consequence of increased lipid accumulation, together with signals derived from extrahepatic organs cooperate to induce inflammation and fibrosis. FFA, free fatty acids; PAMPs, pathogen-associated molecular patterns; ER, endoplasmic reticulum; ROS, reactive oxygen species; HSC, hepatic stellate cell.

NASH is characterized by hepatocellular damage, inflammation and fibrosis [4,5]. In general, simple steatosis is considered a less severe form of NAFLD, although recent data indicate a possible risk of progression [6,7]. In contrast, NASH is a significant risk factor for the development of cirrhosis and hepatocellular carcinoma [8,9,10]. Although NASH was first documented more than 30 years ago [11], its pathogenesis is still not fully elucidated. Initially, a two-hit hypothesis, based on appearance of steatosis (first hit), followed by a second hit leading to inflammation, hepatocyte damage, and fibrosis, was proposed by Day and James [12]. While accumulation of triglycerides is necessary for the development of NASH, they may actually have a protective role against hepatocytes lipotoxicity, which is mainly induced by fatty acids and derived metabolites such as diacylglycerols, acylcarnitines or ceramides [13,14]. In addition, it is still unclear whether NASH develops sequentially, on the grounds of a fatty liver, or it is rather a de novo response to a lipotoxic environment. The multiparallel hypothesis proposed more recently [15] suggests that NASH is the result of numerous conditions acting in parallel, including genetic predisposition, abnormal lipid metabolism, oxidative stress, lipotoxicity, mitochondrial dysfunction, altered production of cytokines and adipokines, gut dysbiosis and endoplasmic reticulum stress. According to this hypothesis, hepatic inflammation in NASH may even precede steatosis. As more contributing factors are continuously identified, a more complex picture of NASH pathogenesis is emerging [16] (Figure 1).

## 2. Genetic Factors

The relevance of genetic factor in the context of NASH has been recently and elegantly outlined by twin studies [17]. A long list of genes potentially implicated in NAFLD appearance and progression has been reported, and these data have been the subject of a recent review [18].

A significant association with a SNP was identified in patatin-like phospholipase domain-containing 3 (*PNPLA3*) on chromosome 22. The variant (rs738409 c.444 C>G, p.I148M), a non-synonymous cytosine to guanine mutation resulting in isoleucine to methionine conversion, correlates with increased hepatic lipid content and predisposes to fatty liver-associated liver disease, from simple steatosis to steatohepatitis, fibrosis and hepatocellular carcinoma [19,20]. *PNPLA3* encodes for a 481 amino acid protein, whose role has not been fully elucidated. It appears to function as acylglycerol hydrolase, acting on triacylglycerol, diacylglycerol, and monoacylglycerol [21,22]. Additional evidence indicates that PNPLA3 also acts as lysophosphatidic acid acetyltransferase [23,24]. Overexpression of the I148M variant in mouse liver promotes accumulation of triacylglycerol, increased synthesis of fatty acids and impaired hydrolysis of triacylglycerol [25]. Moreover, the *PNPLA3* genotype has been reported to influence liver storage of retinol and retinol serum levels in obese subjects [26], suggesting a potential role of PNPLA3 in regulating retinol metabolism and hepatic stellate cell (HSC) biology [27]. Remarkably, PNPLA3 has been recently shown to be expressed in hepatic stellate cells [28]. Interestingly, the prevalence of the *PNPLA3* I148M allele varies considerably among different ethnic groups, with the highest frequency in Hispanics (0.49), and lower frequencies in European Americans (0.23) and African-Americans (0.17) [20]. This is in agreement with the different prevalence of NAFLD in the three ethnic groups.

Carriage of a non-synonymous genetic variant in *TM6SF2* (rs58542926 c.449 C>T, p.E167K) on chromosome 19 (19p13.11) has been reported to correlate with steatosis and increased risk of advanced fibrosis in NAFLD patients [29,30], independently of other factors, including diabetes, obesity, or *PNPLA3* genotype. The minor allele frequency in one of the NAFLD populations tested was 0.12, compared to a frequency of 0.07 in a reference population. TM6SF2, is a transmembrane protein localized in endoplasmic reticulum (ER) and ER–Golgi compartments and functions as a lipid transporter [31]. The amino acid change E167K causes loss of function of TM6SF2 protein. Studies performed in cell lines showed that downregulation of *TM6SF2* reduces lipoproteins and apolipoprotein B (APOB) levels, and increases hepatic deposition of triglycerides and the amount and size of lipid droplets. In contrast, the size and number of lipid droplets diminishes when TM6SF2 is overexpressed, indicating that TM6SF2 plays a role in regulating hepatic lipid efflux [29,31].

A broad spectrum of other genes has been associated with NAFLD. Polymorphism was reported in genes involved in carbohydrate and lipid metabolism, insulin-induced pathways, as well as inflammatory response, oxidative stress and fibrogenesis. A study by Dongiovanni et al. reported that non-synonymous SNPs in ectoenzyme nucleotide pyrophosphate phosphodiesterase 1 (*ENPP1* or *PC1*) (rs1044498, K121Q) and insulin receptor substrate-1 (*IRS1*) (rs1801278, Q972R), are associated with insulin resistance, through impairment of insulin receptor-mediated pathways, such as reduced AKT activation, and promote fibrosis in NAFLD patients [32].

A functional non-synonymous variant (rs1260326, P446L) of glucokinase regulatory protein (GCKR) has also been associated with NAFLD [33]. This variant produces a GCKR with defective inhibitory function, leading to increased glucokinase activity and hepatic glucose uptake [34]. The resultant unimpeded hepatic glycolysis reduces glucose levels, inducing malonyl-CoA synthesis, a substrate for lipogenesis that causes liver fat deposition and impairs mitochondrial β-oxidation. A polymorphism in the solute carrier family 2 member 1 gene (*SLC2A1*), a glucose transporter, has been reported in NAFLD subjects. SLC2A1 downregulation in hepatocytes results in lipid accumulation and oxidative stress [35].

Several genes involved in oxidative stress have been investigated. Two reports correlated the C282Y variant in hemochromatosis gene (HFE) with NASH and higher susceptibility to more severe disease, as fibrosis or cirrhosis [36,37]. However, these findings have not been confirmed by other studies [38,39,40]. Very recently, the rs641738 genotype at the *MBOAT7-TMC4* locus, encoding for the membrane bound O-acyltransferase domain-containing 7 was associated with more severe liver damage and increased risk of fibrosis in patients with NAFLD. This effect has been ascribed to changes in remodeling of the hepatic phosphatidylinositol acyl-chain [41].

## 3. Epigenetics

**Figure 2:**
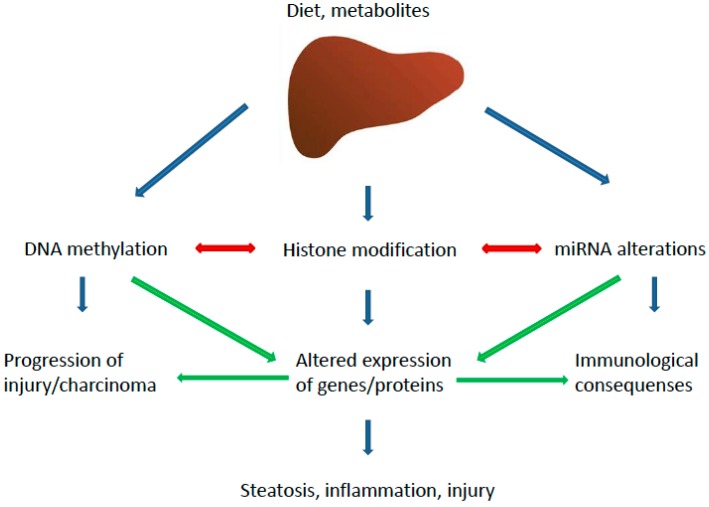
Epigenetic pathways implicated in the pathogenesis of NASH. The major pathways and their main effectors are depicted.

Epigenetic changes consist in modifications at the transcriptional level affecting gene expression and phenotype. A number of epigenetic aberrations have been associated with NAFLD pathogenesis, causing alterations in lipid metabolism, insulin resistance (IR), dysfunction of endoplasmic reticulum (ER) and mitochondria, oxidative stress and inflammation [42]. The different epigenetic pathways potentially involved in NAFLD are summarized in Figure 2.

Aberrant DNA methylation is a major epigenetic process in NAFLD development and progression to NASH [43]. It occurs through methyltransferases (DNMTs) that catalyze the conversion of cytosine to 5-methylcytosine [44], leading to gene silencing. It has been reported that mice fed with a methyl-deficient diet show reduced levels of hepatic *S*-adenosylmethionine (SAM), associated with methylation of genes involved in DNA damage and repair, lipid and glucose metabolism and fibrosis progression [45]. In agreement, food-derived methyl donors, such as folate, betaine and choline, responsible for SAM synthesis, counteract DNA methylation [46] whereas folate deficiency correlates with enhanced fatty acid synthesis and hepatic accumulation of triglycerides (TG) via DNA methylation [47]. Methyl donor supplementation reverts liver lipid deposition induced by high fat/high sucrose-diet, lowering global hepatic DNA methylation and methylation levels of the promoter regions of different regulatory factors [48]. Betaine has been demonstrated to diminish the methylation levels of the promoter of microsomal triglyceride transfer protein (MTP), enhancing hepatic TG export and ameliorating liver steatosis in mice administered a high-fat diet (HFD) [43]. In addition, epigenetic changes of peroxisome proliferator-activated receptor gamma (PPARγ) in the liver of NAFLD patients seems to promote IR [49].

Although most epigenetic alterations are transient, DNA methylation can be inherited from parents [50]. It has been reported that maternal Western diet during prenatal time can increase the susceptibility to NAFLD of male progeny [51]. Novel evidence indicates that mitochondrial DNA (mtDNA) methylation may also play a role in NAFLD pathogenesis [52,53]. Liver methylation of *NADH dehydrogenase 6* (*MT-ND6*) correlates with NAFLD severity, resulting in significantly lower expression of MT-ND6 mRNA in NASH than in patients with simple steatosis [54].

Histone acetylation, regulated by histone acetyltransferases (HATs) and histone deacetylases (HDACs), has been extensively associated with NAFLD [55,56]. High-fat maternal diet was shown to lead to depletion of fetal hepatic HDAC1, suggesting that diet-induced maternal obesity can alter fetal chromatin via histone modifications [55]. Carbohydrate-responsive element-binding protein (ChREBP), an activator of lipogenic and glycolytic pathways involved in NAFLD progression, is regulated by the HAT activator p300. Glucose-activated p300 induces ChREBP hyperacetylation, stimulating its transcriptional activity and hepatic lipogenesis in mice, and p300 overexpression is associated with steatosis and IR [56].

NAFLD has been also correlated with histone methylation. Lipid accumulation in the liver of HFD mice has been associated with H3K4 and H3K9 histone trimethylation of peroxisome proliferator-activated receptor alpha (PPARα) and lipolysis-related genes [57]. In addition, trans-generational changes in histone methylation promote lipogenesis and ER stress, acting on endoplasmic reticulum oxidoreductin 1α (ERO1α) and liver X receptor α (LXRα) [58].

Sirtuins (SIRTs) belong to the silent information regulator-2 family. SIRT1 deacetylation has been recognized as a regulatory mechanism for several proteins involved in NAFLD pathogenesis [59] and low SIRT1 expression has been observed in NAFLD models [60]. In addition, SIRT1-mediated regulation of fetal metabolome and epigenome has been reported under maternal HFD [61]. SIRT3 is localized in mitochondria and regulates fatty acid oxidation. SIRT3 knockout mice fed HFD develop hepatic steatosis and IR [62].

MicroRNAs (miRNAs) modulate gene expression via post-transcriptional mechanisms, regulating the main cellular processes, such as lipid metabolism, inflammation, apoptosis, cell growth and differentiation. In the last few years, aberrant miRNA expression has been reported in a number of diseases including metabolic disorders [63,64], whereas an increasing number of dysregulated miRNAs, implicated in fatty acid synthesis, uptake and storage of triglycerides or oxidation, have been recently identified in NAFLD [65] (Table 1). Among these, miR-122, which negatively regulates hepatic lipogenesis, is reduced in NASH patients [66] whereas miR-34a, that induces β-oxidation and inhibits synthesis of fatty acids via a sirtuin1/5′ adenosine monophosphate-activated protein kinase/3-hydroxy-3-methylglutaryl-CoA reductase (SIRT1-AMPK-HMGCR) mechanism, is upregulated in NAFLD patients [67]. miRNA-33a has been recently reported to participate in NASH development, counteracting cholesterol 7alpha-hydroxylase (CYP7A1). Sterol response element-binding protein 2 (SREBP2) binds to its own gene promoter to induce miR-33a, which leads to a decrease in cholesterol efflux to HDL and bile acid synthesis in Cyp7a1-tg mice [68]. In addition, miR-33a inhibits CYP7A1 and bile acid synthesis to inhibit cholesterol catabolism.

More recently, other dysregulated miRNAs have been identified in NAFLD livers [70]. Among these, the most significantly upregulated (miR-103a-2, miR-106b, miR-576-5p, miRPlus-I137, miR-892a, miR-1282, miR-3663-5p, and miR-3924) play critical roles in insulin signaling, metabolism homeostasis, inflammation and cancer. In particular, miR-576-5p influences multiple pathways implied in NAFLD, including mammalian target of rapamycin (mTOR), a kinase modulated by insulin that induces hepatic lipogenesis through a PPARγ-dependent mechanism [72]. miR-576-5p also regulates eukaryotic translation initiation factor 4 (eIF4), p70S6 kinase (p70S6K) and phosphatidylinositol-4,5-bisphosphate 3-kinase (PI3K), pathways associated with insulin action and metabolic control. Moreover, a direct target of miR-576-5p is the small GTPase RAC1, which promotes lipotoxicity via c-Jun N-terminal kinase (JNK) activation. RAC1 is negatively modulated by miR-576-5p, triggering a protective effect against NAFLD progression [72]. Finally, in a study conducted in biopsy-staged NAFLD patients, increased miR-301a-3p and miR-34a-5p and decreased miR-375 significantly correlated with disease progression [69].

## 4. Dietary Factors

Lifestyle changes focusing on weight loss remain the keystone of NAFLD and NASH treatment [73]. Recent reports indicate that lifestyle modifications based on decreased energy intake and/or increased physical activity during 6–12 months cause improvement in biochemical and metabolic parameters and reduce steatosis and inflammation [74]. Conversely, increased consumption of sugar-sweetened food and beverages has been associated with NAFLD development and progression. High intake of fructose, used as food and drink sweetener, is implicated in NAFLD pathogenesis through several mechanisms. In addition, a fructose-enriched diet contributes to induce liver fibrosis in animal models of NASH [75]. Via the portal vein, dietary fructose reaches the liver in high concentrations, exerting a lipogenic action by activation of the transcription factors SREBP1 and ChREBP and subsequent induction of acetyl-CoA carboxylase (ACC) 1, fatty acid synthase (FAS) and stearoyl-CoA desaturase 1 (SCD1) [76]. These effects persist in liver-specific insulin receptor knockout mice, indicating that fructose stimulates lipogenesis independently of insulin signaling [77]. Fructose-induced de novo lipogenesis (DNL), enhancing malonyl-CoA concentration, inhibits mitochondrial β-oxidation and decreases mitochondrial ATP production [78]. In addition, fructose stimulates lipogenesis by inducing ER stress and subsequently activating the transcription factor X-box binding protein 1 (XBP1), which, in turn, upregulates lipogenic enzymes, as demonstrated in mice fed with a 60% fructose diet [79]. In concomitance, phosphorylation of fructose to fructose-1-phosphate leads to depletion of hepatic ATP and increase in ADP and inosine monophosphate (IMP), which is converted to uric acid [80], that promotes steatosis inducing mitochondrial oxidative stress [81]. Generation of reactive oxygen species (ROS) is also induced by fructose metabolism [82], and nutrient-derived ROS have been associated with enhanced steatosis via insulin-independent PI3K pathway [83]. Moreover, upregulating ketohexokinase, fructose potentiates its own metabolism and ketohexokinase inhibition leads to decreased fatty liver and reduced liver inflammation in high-fat/high-sucrose fed mice [84]. Finally, fructose-induced metabolic disorders can be mediated by epigenetic changes, such as alterations in genomic or mitochondrial DNA (mtDNA) methylation [85,86].

Dietary iron overload has been recently implicated in NASH pathogenesis. A study by Handa et al. [87] shows that dietary iron excess leads to a severe NASH phenotype in an obese, diabetogenic mouse model characterized by oxidative stress, inflammation and ballooning. Different molecular mechanisms are involved, including upregulation of cytokines (interleukin 6, IL-6, tumor necrosis factor α, TNFα) and immune mediators (Toll-like receptor 4, TLR4, inducible nitric oxide synthase, NOS, interferon gamma, IFNγ), and induction of inflammasome related factors (NOD like receptor 3, NLRP3, interleukin 18, IL-18) and genes associated with lipid metabolism. Moreover, emerging evidence indicates that hepatic copper (Cu) deficiency is associated with NAFLD development and progression. In an experimental rat model, a Cu deficient diet coupled with high sucrose intake provoked NASH, even in the absence of obesity or severe steatosis. Rats fed low-Cu/high-sucrose diet displayed enhanced liver expression of lipogenic enzymes, such as ATP citrate lyase (ACLY) and FAS, and of inflammatory and pro-fibrogenic factors (TNFα, C–C motif chemokine CCL2, CCL3), together with hepatic stellate cell activation. While low Cu alone promotes lipid peroxidation, as indicated by increased levels of malondialdehyde (MDA), its combination with high sucrose (or fructose), that causes a further reduction of hepatic Cu, causes insulin resistance and liver damage, with hepatocyte ballooning and occurrence of Mallory-Denk bodies. In addition, Cu deficiency influences Fe retention and partitioning in animals as well as in NAFLD patients [88].

Several lines of evidence correlate hepatic free fatty acids (FFAs) and free cholesterol (FC) accumulation to NAFLD pathogenesis. Dysregulation of lipid homeostasis plays an essential role in NAFLD pathogenesis, induced by a surplus of dietary free fatty acids, enhanced DNL and augmented lipolysis [89]. Rather than total hepatic fat content, the role of specific lipid classes in the development and progression of NAFLD is emerging [90]. In particular, accumulation of different lipids as well as upregulation of distinct enzymes mediating DNL was found to be associated with macrovesicular or microvesicular steatosis, the latter correlating with mitochondrial dysfunction and NAFLD [91]. Among toxic lipids, saturated fatty acids have been shown to be elevated in NASH patients [92] and induce inflammation and hepatocyte apoptosis by activating JNK and mitochondrial pathways. Other lipids having a role in NAFLD include ceramide, diacylglycerol (DAG) and sphingosine [90,93,94,95,96]. In particular, DAG and ceramide impair insulin capability to stimulate glycogen synthesis and suppress gluconeogenesis, through protein kinase-C epsilon (PKCε) activation [97]. In contrast, unsaturated fatty acids do not affect cell viability and an increase in their content leads to enhanced hepatic synthesis of TG. In turn, TG accumulation is not toxic but may protect the liver from the excessive deposition of toxic TG precursors [98,99]. Omega-3 polyunsaturated fatty acids (PUFAs) plasma levels are reduced in patients with NASH. However, pharmacologic supplementation did not induce an amelioration of the histologic picture of NASH [100], and in an experimental model it was even associated with more severe damage [101].

Emerging evidence underscores the role of cholesterol as a prominent risk factor for the pathogenesis of NAFLD/NASH. In humans a progressive increase in hepatic FC during NAFLD progression to NASH has been observed [102,103]. In experimental models increase in dietary cholesterol has been shown to promote hepatic inflammation and fibrosis [104,105,106], whereas a cholesterol-free diet ameliorates NASH [107]. The molecular mechanisms underlying FC accumulation during NASH development are multiple and only partially elucidated. Current data indicate that cholesterol homeostasis is dysregulated in NAFLD, due to an increase in cholesterol synthesis and uptake or dysfunction in cholesterol metabolism. Accordingly, the activity of two key regulators of cholesterol synthesis, HMGCR and SREBP2, is elevated in NASH patients [103,108,109]. Similarly, expression analysis of genes involved in cholesterol metabolism reveals a number of altered pathways in individuals with NASH [108].

Cholesterol uptake from lipoproteins is mediated by different proteins, including the low density lipoprotein receptor (LDLR) and the scavenger receptor class B type I (SR-BI) [110]. Hepatic uptake of LDL-cholesterol occurs via the scavenger receptor pathway in unrestrained manner, leading to deposition of cholesterol crystals in hepatocytes and generation of foamy Kupffer cells, two critical features of NASH [111,112]. Intracellular accumulation of free cholesterol represents a key event for inflammasome activation and inflammatory response [112] and sensitizes cells to transforming growth factor beta (TGF-β), TNF-α and Fas, leading to liver damage and disease progression [104,113]. Moreover, LDL cholesterol can be oxidized to oxidized low-density lipoprotein (oxLDL) cholesterol, which has been found in high concentrations in the plasma of NASH patients [114] and induces proinflammatory cytokine secretion accumulating in lysosomes of Kupffer cells [111,112]. Recently, a reduced efflux of FC has been observed in injured (foam) hepatocytes of NAFLD patients, associated with reduced expression of ATP-binding cassette sub-family G member 8 (ABCG8), which regulates cholesterol excretion trough the bile [108]. In addition, decreased expression of CYP7A1 and CYP27A responsible for cholesterol transformation into bile acids (BA) has been found in human NAFLD/NASH [108], as well as in a rat model of NASH induced by dietary cholesterol overload [115].

Oxysterols, the oxidative products of cholesterol generated during bile acid synthesis, have been described to induce liver damage through mitochondrial impairment. A study by Bellanti et al. [116] shows that mice fed high fat/high cholesterol (HF/HC) exhibit high levels of toxic oxysterols, such as triol, and oxidative stress and mitochondrial dysfunction associated with NASH. Accordingly, Huh7 and primary rat hepatocytes co-exposed to triol and palmitic or oleic acid, undergo apoptosis, mediated by impaired mitochondrial respiratory chain [116]. Finally, besides the effects on liver, cholesterol contributes to NASH pathogenesis also by stimulating inflammatory reactions in other tissues, such as adipose tissue and arterial wall, representing a key factor in the multiparallel scenario concurring to NASH [117,118].

## 5. Mitochondrial Dysfunction and Apoptosis

Oxidative stress has been recognized as a major factor in the pathogenesis of NASH. Based on the evidence that a high amount of intracellular ROS are generated in mitochondria and ROS overproduction is elicited in the presence of respiratory chain disruption, mitochondrial impairment has been suggested as a main event in NASH development [83,119,120]. Along these lines, structural and functional defects in mitochondria have been reported in patients with NASH [121,122].

Several mechanisms contribute to mitochondrial impairment and subsequent hepatic cell injury during NASH, mainly associated with lipotoxicity. It has been shown that, following lipid accumulation, water and calcium influx in mitochondria is increased, due to lower phosphorylation of the voltage dependent anion channel (VDAC) in the mitochondrial outer membrane, resulting in cytochrome c release and cell death [123]. Lipotoxic effects in mitochondria are also mediated by JNK; high concentrations of palmitate cause mitochondrial dysfunction and apoptosis through phosphorylation of Sab (SH3BP5), a mitochondrial outer membrane substrate of JNK [124], whereas free cholesterol accumulation in the liver of NASH mice induces mitochondrial permeability, ROS production and apoptosis through JNK1. An emerging role for NAD^+^ in mitochondrial stress induction during NASH development has been recently shown. Gariani et al. demonstrated that mice fed high-fat/high-sucrose exhibit impaired mitochondrial function associated with lower hepatic NAD^+^ levels [125]. Conversely, NAD^+^ repletion displays a protective effect against NAFLD, probably mediated by the induction of mitochondrial unfolded protein response (UPRmt), an adaptive mechanism dependent on the histone deacetylases SIRT1 and SIRT3, aimed to enhance mitochondrial activity and hepatic β-oxidation [126]. Furthermore, recent studies have suggested a role for coenzyme Q (CoQ), which is essential for mitochondrial respiration, in NAFLD development and progression to NASH [127,128,129,130]. Abnormal concentrations of CoQ have been found in plasma and liver of NAFLD patients [131] and perturbation in CoQ metabolism was observed in experimental NAFLD during disease progression [132,133].

Other key inducers of mitochondrial dysfunction are lysosomal permeabilization, which is frequently observed in NAFLD patients and associated with caspase activation [134], and ROS generation. CYP2E1 promotes oxidative stress, inflammation and protein modifications, by hydrolyzing molecules such as fatty acids and ethanol into toxic metabolites, including ROS, which cause respiratory chain disruption and mitochondrial damage [135], resulting in hepatocyte injury and progression to NASH [136].

## 6. Necroptosis

Necroptosis is a recently described cell death mechanism, morphologically comparable to necrosis, but consisting in definite biochemical pathways that occur in a programmed mode [137] and are potentially involved in inflammatory disorders, including liver diseases. Necroptosis can be initiated by activation of multiple signals, such as toll-like receptors, death receptors and others, which lead to the assembly of the necrosome, a multiprotein complex consisting in caspase-8, Fas-Associated protein with Death Domain (FADD), cellular FLICE/caspase 8-like inhibitory protein (cFLIP), and receptor-interacting proteins 1 and 3 (RIP1 and RIP3) [138]. RIP1–RIP3 interaction initiates necroptotic signaling [139]; RIP3 phosphorylates mixed lineage kinase domain-like protein (MLKL), which oligomerizes and translocates to the plasma membrane causing irreversible membrane damage and consequent cell death [140]. In specific cell setting, RIP3 can mediate necroptosis independently of RIP1 [141,142,143]. In other cell contexts, a RIP3 dependent ROS production may play an additional role [144,145].

Recently, necroptosis has been proposed as a novel mechanism in the pathogenesis of NAFLD both in humans and experimental models. Gautheron et al. found that RIP3 was overexpressed and mediated liver inflammation, activation of hepatic progenitor cells/cholangiocytes and liver fibrosis in NASH patients and in the methionine/choline-deficient (MCD) mouse model of steatohepatitis. They observed that RIP3 induces JNK activation, leading to release of pro-inflammatory mediators, such as CCL2, that further sustain RIP3-dependent signaling, cell death, and liver fibrosis. RIP3-induced pathways were blocked by caspase-8. [146]. A study by Afonso et al. [147] confirmed that hepatic levels of RIP3 are significantly augmented in steatohepatitis and showed that RIP3-dependent MLKL activation is increased in the liver of NAFLD patients as well as in MCD-induced experimental NASH. Moreover, lack of RIP3 ameliorates liver injury, steatosis, inflammation and fibrosis in experimental NASH.

## 7. Endoplasmic Reticulum Stress

ER stress has been implicated in a number of liver diseases, including NASH. ER dysfunction, ATP depletion or other stimuli induce the unfolded protein response (UPR), an adaptive mechanism directed to avoid luminal accumulation of defective proteins and apoptosis initiation. In NAFLD, a cross-talk between insulin signaling and UPR has been reported, involving XBP–1/PI3K interaction and consequent XBP-1 nuclear translocation [148]. Other pathways activated by cellular response to ER stress involve JNK, an activator of inflammation and apoptosis implicated in NAFLD progression to NASH [90] and SREBP-1c, which induces liver fat accumulation, worsening ER stress [149]. In vitro studies show that exposure of hepatic cells to a lipotoxic concentration of palmitate, a saturated fatty acid (SFA), is associated with ER calcium depletion, ROS accumulation and apoptosis [92,150,151]. In fact, increased SFA incorporation in ER membrane, as well as altered phosphatidylcholine/phosphatidylethanolamine ratio, induces disruption of ER membrane and impairment of sarcoendoplasmic reticulum calcium ATPase (SERCA) function, causing a net calcium efflux from ER stores and its subsequent translocation to the mitochondria, with dysregulation of mitochondrial metabolism and oxidative stress. Accordingly SERCA activity is impaired in obese livers [152] and overexpression of SERCA in obese mice improve hepatic ER stress, indicating that SERCA plays a crucial role in lipotoxic-induced ER stress and, indirectly, in mitochondrial dysfunction [152].

## 8. Hypoxia

In experimental NASH, hypoxia causes alterations in lipid homeostasis, upregulating genes involved in lipogenesis, such as SREBP-1c, PPARγ, ACC1 or 2 and downregulating genes implied in lipid metabolism, such as PPARα and carnitine palmitoyltransferase-1 (CPT-1) [153]. Besides lipid metabolism, insulin signaling is also affected and under hypoxic conditions hepatic upregulation of inflammatory cytokines and profibrogenic genes was observed [154]. Moreover, reduced oxygen availability induces secretion of adipokines and inflammatory cytokines in adipose tissue [155], contributing to alter lipid metabolism and glucose homeostasis [156,157]. These effects are mediated by hypoxia-inducible transcription factors (HIF-1α and HIF-2α) that regulate cellular response to oxygen deficiency and can be also activated by other stimuli, including oxidative stress or inflammatory signals [158]. In particular, HIF-1α transcription is induced by nuclear factor kappa-B (NF-κB), and NF-κB activity is crucial for HIF-1α accumulation under oxygen deprivation [159]. Furthermore, hypoxia has been reported to modulate inflammation by regulating TLR expression and function through HIF-1 [160,161]. Along these lines, it is conceivable that the proinflammatory state observed in obese NAFLD patients may be enhanced by hypoxia, due to a positive feedback mechanism involving HIF-1α and NF-κB, explaining the exacerbation of liver injury in NAFLD subjects in the presence of obstructive sleep apnea-hypopnea syndrome (OSAHS) [162].

## 9. Inflammation

Inflammation represents a crucial aspect in NASH pathogenesis. Overload of toxic lipids, mainly FFA, causes cellular stress and induces specific signals that trigger hepatocyte apoptosis, the prevailing mechanism of cell death in NASH, correlating with the degree of liver inflammation and fibrosis [163]. Signaling pathways induced by key death receptors, such as TNF-related apoptosis-inducing ligand (TRAIL-R), Fas and tumor necrosis factor receptor (TNFR), are upregulated in NASH, indicating they may have a role in promoting inflammation and chemokine secretion. Although the precise role of Fas and TNFR in NASH in vivo is still controversial, it has been shown that lack of TRAIL-R is protective, as TRAIL-R-deficient mice display reduced steatosis, inflammation and fibrosis in association with lower hepatocyte apoptosis [164]. Moreover, prolonged ER stress and mitochondrial dysfunction, two critical events in NAFLD, have been reported to induce apoptosis through TRAIL-R/caspase 8 [165].

Different types of immune cells are recruited and/or activated to the site of injury, contributing to NAFLD development and progression. Kupffer Cell (KC) activation is critical in NASH and precedes the recruitment of other cells [166]. Lanthier et al. [167] have shown that KC depletion increases insulin sensitivity and ameliorates inflammation and fibrosis. Depending on the settings, different polarization forms have been described for KCs, mainly classified in two phenotypes: M1, pro-inflammatory and M2, considered primarily immunoregulatory [168]. However, markers of both M1 and M2 forms can be expressed at once [169]. Differentiation of KCs towards a M1 phenotype is principally driven by pathogen-associated molecular patterns (PAMPs) that, interacting with TLRs, induce the secretion of various cytokines, such as IL-1β, IL-12, TNF-α, CCL2 and CCL5, concurring to further hepatocyte damage and release of damage-associated molecular patterns (DAMPs). DAMPs, in turn, act on TLRs amplifying KCs activation and inflammation. In addition, some cytokines (i.e., CCL2 and CCL5), induce HSC activation, initiating a fibrogenic response [170]. Activation of KCs in NAFLD is also triggered by toxic lipids, that upregulate TLRs and augment the response to lipopolysaccharide (LPS) [171]. KCs displaying the M2 phenotype produce several factors with anti-inflammatory properties, as IL-4, IL-10, IL-13 and TGF-α [168,169], but different subtypes have been identified with diverse actions. Although it has been reported that induction of peroxisome proliferator-activated receptor delta (PPARδ) drives KCs toward the M2 form, reducing obesity-induced insulin resistance in mice [172], the role of M2 KCs in NAFLD is still not elucidated [168].

Despite potent antimicrobial and phagocytic properties, neutrophils display scarce specificity. Excess of neutrophil recruitment in NASH crucially contributes to hepatocyte damage, inflammation and fibrosis, through the release of different factors [173,174], including cytotoxic enzymes as myeloperoxidase and elastase. Myeloperoxidase-deficient mice show moderated NASH, associated with lower hepatic secretion of inflammatory cytokines [175]. Similarly, deletion of neutrophil elastase attenuates liver inflammation in experimental NAFLD [176].

Dendritic Cells (DCs) counteract sterile inflammation acting as antigen-presenting cells and eliminating cell debris and apoptotic cells. Studies aimed to establish DCs’ function in NASH have shown controversial results [177]. An anti-inflammatory and antifibrotic role of DCs in NASH is suggested by the fact that liver depletion of these cells exacerbates inflammation and fibrosis. According to the study by Henning et al liver infiltrating DCs activate and secrete IL-6, TNF-α and CCL2 [178]. In contrast, other findings report that avoiding the accumulation of DCs subtypes expressing high levels of inflammatory factors limits liver injury in experimental NASH [179].

Natural Killer (NK) cells in the liver are stimulated through several receptors upon interaction with other hepatic cells. In NASH, activation of NK can be achieved by a broad number of ligands and cytokines, but the role of these cells in NAFLD pathogenesis is still controversial [180,181]. Two different phenotypes of NKT cells have been recently associated with liver disease, acting in opposite modes during sterile inflammation: proinflammatory type I and protective type II cells [182]. Although NKT type I cells can be activated by lipids, suggesting their possible involvement in NAFLD, NKT-deficient mice fed HFD are more prone to steatosis and weight gain than wild type mice [183]. In addition, adoptive transfer of NKT cells in leptin-deficient mice ameliorates glucose metabolism and diminishes fatty liver [184]. Furthermore, depletion of NKT can result in activation of KC and secretion of IL-12 [184]. Conversely, clinical studies performed in patients with different stages of NAFLD demonstrate that NKT cells tend to increase in the liver during disease progression [185]. According to these data, NKT cells seem to be depleted in early NAFLD to enhance in the later phases, participating in inflammation and fibrosis [186].

## 10. Hedgehog

Hedgehog (Hh) is a well-characterized factor implied in the fibrogenic process of several organs, including the liver. Hh pathway activation is proportional to the severity and persistence of injury [187], induces a cascade of events concurring to wound healing response and involves various cell types, including damaged ballooned hepatocytes, inflammatory cells (mainly NKT cells and macrophages), ductular/progenitor cells and HSCs [188].

The Hh pathway was associated with severe NASH in a gene profiling study where patients with different severity of the disease were included [189]. In experimental NASH, the Hh pathway leads to proliferation and activation of ductular progenitor cells and HSC, that, in turn, produce Hh ligands and, consequently, soluble mediators such as osteopontin and CXCL-16, responsible for immune cells recruitment and damage progression [190,191]. Moreover, Patched-heterozygous deficient mice, characterized by hyperactivation of the Hh pathway, show exacerbation of the disease following a NASH-inducing diet, whereas liver-specific inhibition of Smo prevents diet-induced liver damage and fibrosis, despite hepatic lipid accumulation [190].

Caspase-2 has been recently identified as a critical factor in NASH pathogenesis, mediating hepatocyte lipoapoptosis. Hepatic caspase-2 was found to be increased both in human and experimental NASH, in association with profibrogenic factors, such as Hh-related genes. When challenged with a HF diet or fed a MCD diet, caspase-2 knockout mice showed lipid-induced hepatic apoptosis, together with decreased activation of Hh signaling and fibrosis [192].

In NAFLD patients, Hh activity and Hh ligands’ expression correlates with the degree of fibrosis [193] and elevated Hh activation is associated with hepatocyte ballooning, high presence of progenitor cells and myofibroblasts and portal inflammation [187]. In agreement with these findings, the Pioglitazone vs. Vitamin E vs. Placebo for treatment of NASH (PIVENS) trial demonstrated that amelioration of NASH in response to treatment was associated with a marked decrease of Sonic Hh ligand (Shh) expressing hepatocytes [194].

## 11. Nuclear Receptors

Nuclear receptors are ligand-dependent transcription factors that regulate glucose and lipid metabolism in the liver. Nuclear receptors are divided into seven subfamilies named as NR0-NR6 [195] and NR1 subfamily is of particular importance in NAFLD. This latter group of nuclear receptors is retained in the nucleus and heterodimerizes with the retinoid X receptor (RXRα) [195,196] and includes: NR1C1-3 (the peroxisome proliferator-activated receptors, PPARα, β, γ), NR1H2-3 (the liver X receptors, LXRα, β), NR1H4 (the farnesoid X receptor, FXRα), NR1I2 (the constitutive androstane receptor, CAR), and NR113 (the pregnane X receptor, PXR). PPARs inhibit inflammation in the obese state acting on NF-κB and AP1 transcription factor and regulate metabolism by inducing transcription of adiponectin (PPARγ) and fibroblast growth factor-21 (FGF21) (PPARα and FXR) [195]. PPARα regulates β-oxidation and cholesterol removal during the fasting state or when metabolism increases in adipose and/or muscle tissues [195]. Hepatic PPARα expression decreases in NAFLD leading to steatosis, but is enhanced following diet and exercise [197,198].

In animal models of steatosis and steatohepatitis, the use of PPARα activators improves the disease [199,200]. In addition, several studies in mice suggest that induction of both PPARβ/δ and PPARγ ameliorates steatosis [201,202]. Indeed, animals treated with PPARα activators show less weight gain than controls, lower levels of epididymal fat, and are protected from atherosclerosis [203]. PPAR activation may also ameliorate fibrosis, since NASH patients treated with pioglitazone (a PPARγ agonist) had improved fibrosis biomarkers [204]. Recent studies show that PPARγ downregulates adipocyte endothelial nitric oxide synthase (eNOS), a molecule that contributes to IR and development of NASH [205]. Since the use of selective PPARα agonists has proven quite ineffective against NAFLD [197] the use of mixed receptor agonists (PPARα and PPARβ/γ) is underway in the therapy for NASH patients and recent results have been reported [206].

PXR, expressed in many tissues but mainly in the liver [207], is released not only by hepatocytes, but also by Kupffer and stellate cells [208]. Two polymorphisms of this gene have been associated with augmented severity of NAFLD: rs7643645/G and rs2461823 [209], whereas a variant encoding a short dominant negative PXR isoform, which inhibits the full-length isoform activity, has been recently described [210]. PXR regulates various genes involved in xenobiotic and drug metabolism, including enzymes [211] that play a role in the oxidative metabolism of lipophilic compounds such as steroids, fatty acids, bile acids, drugs, retinoids, and xenobiotics. PXR activation has been associated with increased severity of steatosis, obesity, insulin resistance and hypercholesterolemia as it enhances hepatic fatty acid uptake and lipogenesis, while it decreases β-oxidation [212,213]. The role of PXR in experimental NAFLD is more complex. While PXR knockout mice are resistant to obesity, they show impaired glucose tolerance, hyperleptinemia and hypoadiponectinemia, together with elevated fasting glucose levels [212]. Recently, it was shown that PXR activation inhibits the production of many NF-κB target genes and increases the production of secreted interleukin-1 receptor antagonist (IL-1RA), reducing the effects of LPS-induced inflammation [214].

Human CAR1-3, expressed mainly in liver and intestine and to a lower extent in other tissues [215], is implicated in protection against toxic food or contaminants [216]. CAR is also associated with lipid metabolism and inflammation in NAFLD. CAR increases in the liver in the fed state, reducing hepatic steatosis, inflammation, insulin resistance and hypercholesterolemia [217]. In animal models, treatment with an agonist of CAR ameliorates diet-induced obesity, hepatic steatosis and diabetes [218]. Moreover, in knockout mice for the low density lipoprotein receptor (LDLR), activation of CAR reduces triglycerides and cholesterol plasma levels [219]. Recently, it has been reported that activated CAR translocates into the nucleus and functions as an adaptor protein to recruit PGC1α to the Cullin1 E3 ligase complex for ubiquitination. The interaction between CAR and PGC1α also induces the degradation of PGC1α and suppression of gluconeogenesis both in vitro and in vivo [220]. CAR can induce carcinogenesis in mice, although this effect has not observed in humans [221]. Indeed, CAR activation in humans may have antiproliferative effects, as demonstrated by a recent report showing that CAR-deficient HepaRG cells have increased expression of proliferative genes [222].

FXR, highly expressed in liver, kidney, intestine, and adrenals, inhibits the expression of CYP7A1 and sterol 12-α-hydroxylase (CYP8B1), genes involved in bile acid synthesis from cholesterol. Besides its central role in bile acid metabolism, FXR activation also regulates the expression of various genes involved in glucose, lipid, and lipoprotein metabolism, crucial in NAFLD [223]. Hepatic FXR inhibits fatty acid synthesis and uptake and upregulates beta oxidation, regulating lipid homeostasis [224]. In NAFLD patients and in animal models, activation of FXR by obeticholic acid (OCA) decreases both steatosis and obesity [225,226]. In HF/HC diet-treated mice, the FXR agonist GW4064 decreased the expression of the hepatic lipid transporter CD36, reducing hepatic steatosis and weight gain [227]. FXR can regulate insulin resistance as recently demonstrated in NASH patients treated with OCA, which improves insulin sensitivity [228]. Similarly, OCA treatment in Zucker (fa/fa) rats improves insulin sensitivity, and GW4064 treatment, in HF/HC diet mice, reduces hyperinsulinemia and hyperglycemia [226]. Besides OCA and GW4064, further potential novel therapeutic targets in NASH are currently in phase II clinical development [229].

Intestinal activation of FXR reduces weight gain, liver glucose production and steatosis, stimulating human fibroblast growth factor-19 (FGF19). This factor inhibits CYP7A1 resulting in an inhibition of liver bile acid synthesis. Indeed, administration of FGF19 in mice and rats animal models increases fat oxidation and decreases liver triglycerides and glucose levels [230,231]. Recent studies show that activation of intestinal FXR by feraxamine inhibits weight gain induced by diet, hepatic glucose production and steatosis. These effects are mediated by fibroblast growth factor-15 signaling, without interfering with hepatic FXR activation [232]. Intestinal FXR agonism promotes adipose tissue browning and reduces obesity and insulin resistance, suggesting that tissue-specific activation of FXR may be a novel approach to treat NAFLD. Activation of intestinal FXR affords hepatoprotection by restoring hepatic homeostasis, regulating cellular proliferation and decreasing hepatic fibrosis and inflammation [233].

## 12. Pattern Recognition Receptors and the Inflammasomes

Toll-like receptors are highly conserved receptors that recognize endogenous danger signals, such as molecules released by damaged cells (damage-associated molecular patterns, DAMPs) or exogenous danger signals, as gut-derived pathogen-associated molecules (pathogen-associated molecular patterns, PAMPs) [234,235]. Due to the high liver exposure to danger signals via the portal system, TLR-induced pathways play a central role in activation of hepatic cells, primarily Kupffer cells, but also hepatocytes and HSC. As pattern recognition receptors (PRR), TLRs act as defense mechanism, but are also implicated in the pathogenesis of NASH [236,237]. Among NAFLD-related TLRs, TLR2 interacts with a broad range of PAMPs, including peptidoglycan, a surface component of Gram-positive bacteria [238], which appears to be increased in NAFLD [239]. Importantly, inhibition of TLR2 signaling prevents insulin resistance in HFD mice [240], whereas TLR2-deficient mice fed HFD display reduced levels of inflammatory cytokines and do not develop NASH [241].

The role of TLR5 in NAFLD pathogenesis is still unclear, as only a correlation with dysbiosis and metabolic syndrome has been reported [242,243]. TLR9, an intracellular receptor, is activated by unmethylated DNA, typically express in viruses and bacteria but rare in mammalian cells. TLR9 downstream signaling involves IL-1, and is associated with NASH severity and fibrosis [244]. A study conducted in an experimental model of colitis, with high portal levels of LPS, shows increased TLR9 liver expression, associated with hepatic steatosis, inflammation, and fibrosis [245].

The crucial role of TLR4 in NAFLD pathogenesis has been demonstrated in TLR4-deficient mice, that display lower levels of inflammatory mediators and fail to develop NAFLD or insulin resistance [246]. TLR4 plays a major role in linking innate immunity with inflammatory response and the function of TLR4 in Kupffer cells is well characterized [247]. TLR4 is primarily activated by Gram-negative bacterial lipopolysaccharides (LPS), leading to overexpression of cytokines, chemokines and antimicrobial molecules [248,249]. LPS/TLR4 interaction, that requires LPS-binding protein and two co-receptors (CD14 and myeloid differentiation protein 2, MD2), activates downstream pathways in a myeloid differentiation factor (MyD)88-dependent or independent fashion [250]. The MyD88-dependent pathway signals through IκB kinase (IKK)/NF-κB and mitogen activated protein kinase (MAPK)/AP-1, inducing the expression of pro-inflammatory cytokines (TNF-α, IL-1β, IL-6 and IL-12) and genes implicated in the immune response [250]. The MyD88-independent cascade involves IFNs [250]. ROS production and subsequent activation of the unfolded protein response are also induced in TLR4-activated Kupffer cells, representing an additional mechanism triggered by TLRs in NAFLD progression [251].

Besides Kupffer cells, TLR4 is expressed by other hepatic cells, including HSCs, hepatocytes and cholangiocytes and LPS/TLR4 axis plays a critical role in the pathogenesis and progression of fatty liver diseases, as demonstrated by increased levels of portal endotoxins and TLR4 hepatic expression in experimental NASH [252,253]. Based on its expression in HSC, a direct role of TLR4 in liver fibrogenesis has been suggested. According to this hypothesis, the expression of chemokines and adhesion molecules, as well as TGF-β-mediated signaling, are positively modulated by TLR4 [254], while two TLR4 polymorphisms, protective against fibrosis, are associated with a lower apoptotic threshold for HSC [255].

**Figure 3:**
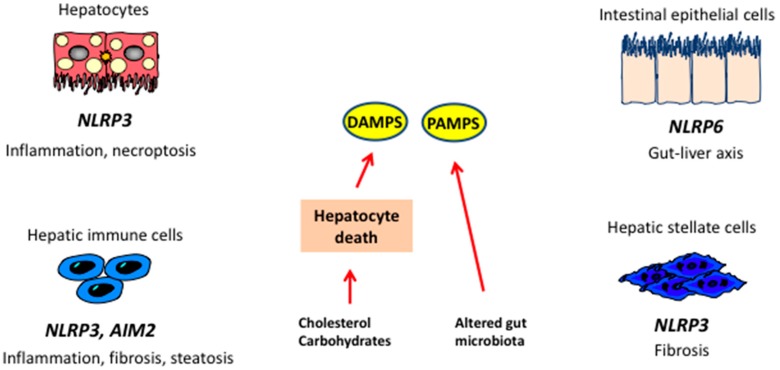
Inflammasomes and the liver. In steatosis, hepatic damage leads to generation of damage-associated molecular pattern (DAMPs), while alterations in microbiota lead to increased availability of pathogen-associated molecular patterns (PAMPs). DAMPs and PAMPs act on receptors localized on liver cells leading to activation of different inflammasomes and release of cytokines implicated in NASH. NLRP3: NOD-like receptor family, pyrin domain containing 3; AIM2: Abscent in melanoma 2.

TLR4-mediated inflammatory response can also be elicited by DAMPs released by necrotic cells, such as high mobility group box 1 (HMGB1) or phospholipids. These molecules stimulate monocyte and Kupffer cells to secrete inflammatory mediators (Figure 3). It is noteworthy that, in the presence of high glucose, TLR4 activation and downstream signaling can be triggered by FFA [256], clarifying, at least in part, the mechanism by which saturated fatty acids, frequently enhanced in plasma of obese patients, have toxic effects [257].

An important role in NASH pathogenesis has been recently ascribed to the nucleotide oligomerization domain (NOD)-like receptors (NLRs). NLR activation in response to DAMPs or PAMPs leads to the assembly of inflammasome, a multiprotein complex required for caspase-1 activity and initiation of inflammatory signals. Full activation of inflammasome, mediated by PRRs via NF-κB, can be induced by a broad spectrum of signals, such as uric acid, ROS, ATP [258] and mitochondrial DNA [259], and results in secretion of mature IL-1 and IL-18 [260,261]. These cytokines, acting on different cell types, elicit inflammatory signals in liver as well as in the adipose tissue and intestine, triggering steatosis, insulin resistance, inflammation and cell death [262]. A role for inflammasomes in NAFLD development and progression to NASH has been shown both in humans and animal models [263,264]. Activation of NLRP3 inflammasome has been reported in MCD diet-induced steatohepatitis [265], as well following protracted HF/HC/HS feeding [266]. Moreover, NLRP3 gain of function correlates with liver fibrosis. Conversely, absence of this receptor appears to improve metabolic activity [267] and diet-induced steatohepatitis [268], although a study by Henao-Mejia et al. [269] demonstrated that lack of NLP3 promotes gut dysbiosis and chronic inflammation. Activation of NLRP3 inflammasome has been associated with hepatocyte pyroptosis, a recently described, inflammasome-mediated cell death mechanism [268,270].

Hepatocyte damage leads to secretion of intracellular molecules, DAMPs, acting as danger signals capable to recruit and/or activate immune cells and initiate an inflammatory response in the absence of pathogens, a mechanism referred as sterile inflammation [271,272]. Several DAMPs have been identified, including nuclear and mitochondrial DNA, purine nucleotides (ATP, UTP), nuclear factors as HMGB1 and uric acid [180,273]. Besides mitochondrial DNA, which activates TLR9, a number of mitochondrial components have been shown to play a part in sterile inflammation [274,275], including formyl-peptides, ATP and ROS, that act by inducing inflammasome activation [276,277,278]. High concentrations of extracellular ATP, as a consequence of cell death, result in inflammasome activation and IL-1β production, via P2X7 receptor [279]. As binding of ATP to P2X7 provokes pore formation in the plasma membrane, allowing bacterial products to enter the cells, ATP plays a role also in pathogen-associated molecular pattern-induced inflammation [280].

HMGB1 is a constitutively expressed nuclear protein that induces transcriptional activation [281], and is released in response to different stimuli, such as PAMPs and DAMPs [282,283]. HMGB1 interacts with a broad spectrum of receptors (TLR4, TLR2, TLR9, and RAGE) exerting proinflammatory actions in complex with other factors, as single stranded DNA, LPS and IL-1β [284].

In its crystal form, uric acid induce inflammatory response by inflammasome activation in a receptor-independent manner, causing phagosome burst and spill of cytosolic proteases [285]. In some settings, DAMPs can be also secreted independently of apoptosis. HMGB1 production can occur by activated macrophages in response to LPS, TNF, and TGFβ [286]. Moreover, secondary necrosis, due to impaired efferocytosis, may contribute to release of intracellular components amplifying the inflammatory response.

## 13. Adipokines

Adipose tissue is recognized as an endocrine organ that secretes adipokines, which are peptides with autocrine, paracrine and endocrine functions, controlling systemic metabolism and energy homeostasis [287]. Among these, leptin and adiponectin are involved in the pathogenesis of NAFLD and progression to NASH, leptin being identified as a profibrogenic adipokine [285,288]. Adipose tissue also produces other molecules (including classical cytokines), mostly released by endothelial or immune cells, such as TNF-α and IL-6 [289]. Adiponectin has in general a beneficial impact on NAFLD [290], while others, as resistin, TNF-α and IL-6 possibly have an adverse impact. In particular, adiponectin reduces IR and shows anti-steatotic and anti-inflammatory properties, while TNF-α increases IR and displays pro-inflammatory effects [291,292]. In physiologic conditions, cytokine-adipokine interplay is finely regulated, but in some setting, such as increased adipose tissue mass, the critical balance between cytokines and adipokines is compromised, leading to chronic inflammation, IR and NAFLD [292]. Leptin, an adipokine which plays a major role in energy homeostasis, is mainly produced by adipose tissue, but it is also synthesized in other organs [293]. Consequent to an increase in adipose tissue mass, leptin is upregulated, acting as compensatory factor in preserving insulin sensitivity and exerting anti-steatotic effects. Nevertheless, if adipose tissue continues to augment, the compensatory mechanism fails, with a sustained rise in IR and hepatic steatosis [294]. Leptin-mediated dual action has been demonstrated in experimental NAFLD, as in early disease leptin exerts a protective effect by inhibiting hepatic glucose production and de novo lipogenesis through stimulation of fatty acid oxidation, while as NAFLD proceeds, it acts as a pro-fibrogenic and inflammatory factor [294]. Novel evidence indicates that leptin-mediated nicotinamide adenine dinucleotide phosphate (NADPH) oxidase increases the levels of miR21, which is a key regulator of TGF-β signaling. The rise in miR21 increases TGF-β and SMAD2/3-SMAD4 nuclear colocalizations, whilst repressing SMAD7 [295]. In addition, leptin reduces PPAR-γ expression in HSCs, promoting hepatic fibrosis [296]. A recent study, conducted by Heinrich et al., shows that leptin resistance contributes to obesity in null mice mutated for carcinoembryonic antigen cell adhesion molecule 1 (CEACAM1) [297]. CEACAM1 is a molecule that induces insulin clearance [298] and reduces fatty acid synthesis in liver in the presence of insulin resistance, hepatic steatosis and visceral obesity [299]. Furthermore, (Cc1^−/−^) mice develop hyperleptinemia, firstly related to the augmented visceral obesity, followed by hyperphagia and reduced physical activity. These effects are possibly due to leptin resistance and elevated hypothalamic fatty acid synthase activity, that could, in turn, be mediated by both central and peripheral factors [297].

Adiponectin is one of the most abundant adipokines, and is also produced by hepatocytes in response to liver injury [300]. It exhibits anti-steatotic and antiapoptotic actions on hepatocytes and exerts anti-inflammatory and anti-fibrotic effects acting on HSC, Kupffer and sinusoidal cells [301]. Adiponectin amounts drop when adipose mass increases, but the underlying mechanism is not completely elucidated. It may involve adipose tissue hypoxia, oxidative stress [155,302] and increased inflammatory mediator levels [303]. Another potential factor linking adipocyte hypertrophy to reduced adiponectin synthesis is mitochondrial dysfunction [304]. Recent reports show that 11β-hydroxysteroid dehydrogenase type1 (11β-HSD1) expression increases in hypertrophic adipocytes and this could be responsible for mitochondrial dysfunction and reduced adiponectin synthesis.

After NASH progression towards cirrhosis, circulating adiponectin seems to increase [305], probably due to two main mechanisms: a decrease in hepatic clearance of adiponectin and/or a compensatory mechanism aimed to buffer the hyper-secretion of inflammatory cytokines. Recent studies show that in the compensated late stage of NASH, circulating adiponectin is associated with hepatic lipid loss [306]. These data reinforce the theory that adiponectin may be involved in the “burnt-out NASH”, characterized by the loss of hepatic lipids, often observed in advanced fibrosis and cirrhosis.

Adipose tissue (mainly visceral) and liver (mainly hepatocytes) are the principal producers of chemerin [307]. Chemerin concentrations, which are generally higher in obesity and IR and drop after weight loss, may modulate insulin resistance and inflammatory responses [308]. Animal models of obesity and IR (*ob*/*ob* and *db*/*db* mice) display increased chemerin expression [309,310]. A recent study conducted in NAFLD subjects show that circulating levels of chemerin positively correlate with body mass index (BMI) and are also higher in individuals with impaired glucose tolerance (IGT) or type 2 diabetes. In MCD-induced NASH, hepatic levels of chemerin tend to increase. In human NASH, liver chemerin mRNA is upregulated in respect to healthy controls, but similar levels have been found also in steatosis [311].

## 14. Microbiota

Accumulating evidence indicates that dysregulation of microbiota components are involved in various liver diseases, including NAFLD and NASH, through obesity predisposition, metabolic alterations and liver inflammation. Gut microbiota produces extra energy for the host, processing polysaccharides to short-chain fatty acids (mainly acetate, propionate, and butyrate) [312] and stimulating lipogenesis. A potential role of specific gut microbiome has been suggested in the pathogenesis of NAFLD, as obese mouse models host 50% less *Bacteroides* and more *Firmicutes* compared to lean control [313], and germ-free mice show significantly greater increase in body fat following colonization with an “obese microbiome” [313]. Conversely, a recently described bacterium, *Akkermansia muciniphila*, has been associated with a non-obese phenotype both in humans and animal models, and HFD mice administered with *Akkermansia* show reduced adipose tissue inflammation and increased glucose tolerance [314,315]. The intestinal microflora produces enzymes that metabolize dietary choline, a cell membrane component regulating lipid transport in liver, into methylamines, toxic compounds responsible for inflammation and liver injury [316]. Aberrant microbiota could induce triglyceride accumulation and promote NASH both reducing choline and increasing methylamines [317].

Alterations in bile acid metabolism have been reported during NAFLD development. Intestinal bacteria can modify bile acid pool through the conversion of cholic and chenodeoxycholic acid into secondary bile acids, influencing lipid and glucose homeostasis. In addition, abnormal microbiota can impair bile acid receptor signaling, such as FXR and the G-protein-coupled bile acid-activated receptor TGR5 [318,319], affecting hepatic de novo lipogenesis and very low-density lipoprotein VLDL export [320] as well as glucose metabolism [321,322].

Endogenous ethanol is produced by several microbiome species. Ethanol induces hepatotoxicity stimulating Kupffer cells to produce nitric acid and cytokines, whereas ethanol metabolites promote triglyceride accumulation and oxidative stress in the liver. In addition, ethanol impairs gut mucosal permeability inducing endotoxemia. Enhanced breath ethanol content was found in *ob*/*ob* mice and it was abolished by antibiotic treatment [323]. Increased ethanol levels were also detected in obese individuals and in children with NASH [324].

The gut microflora plays an important role in the development and function of the host immune system [325]. Through the portal circulation, liver is directly exposed to gut-derived products, being the first line of defense against bacterial toxins. Enhanced levels of circulating LPS and endotoxins have been detected in rodents with diet-induced NAFLD and in NASH patients, respectively. LPS, the active component of endotoxins, interacts with LPS-binding protein and the CD14 receptor, activating TLRs and, consequently, the inflammatory cascade that involves stress-activated protein kinases, JNK, p38, interferon regulatory factor-3 (IRF-3) and NF-κB, pathways implicated in insulin resistance and triglycerides synthesis [252,325].

Finally, a correlation between small intestinal bacterial overgrowth (SIBO) and NAFLD has been observed in clinical and experimental studies [237,326,327]. Bacterial overgrowth in the small intestine, as well as qualitative microbiome abnormalities can impair the barrier functions of the intestinal mucosa, leading to enhanced mucosa permeability and subsequent translocation of endotoxin to the bloodstream [328,329]. Therefore, increased gut permeability represents an additional mechanism in NASH pathogenesis, acting through the accumulation of endotoxin and bacterial metabolites in liver and subsequent induction of inflammatory responses, via activation of pattern recognition receptors.

## 15. Perspectives

Extensive information has accumulated in the past few years on the molecular mechanisms underlying the development of steatohepatitis. This has been paralleled by a number of clinical trials exploring novel approaches, in part derived from preclinical data. Continuing research in this field will be instrumental in providing new targets and biomarkers for the management of this very prevalent condition.

## Figures and Tables

**Table 1:** Modulation of miRNA expression relevant to NAFLD/NASH. *Δ* indicates up- (↑) or downregulation (↓). CYP7A1: cholesterol 7α1-hydroxylase; SREBP2: sterol response element-binding protein 2; SIRT1: sirtuin1; AMPK: 5′ adenosine monophosphate-activated protein kinase; HMGCR: 3-hydroxy-3-methylglutaryl-CoA reductase; FAS: fatty acid synthase; ACC: Acetyl-CoA carboxylase; mTOR: mammalian target of rapamycin; ROS: reactive oxygen species; RAC1: Ras-related C3 botulinum toxin substrate 1; ?: mechanism and/or target unknown.

miR	*Δ*	Disease	Model	Role	Validated/Predicted Target	Reference
33a	↑	NASH	Mouse Liver	Cholesterol and bile acid homeostasis	CYP7A1, SREPB2	[68]
34a	↑	NAFLD/NASH	Human Biopsies	Lipid homeostasis	SIRT1-AMPK-HMGCR	[67,69]
103a2	↑	NAFLD	Human Biopsies	Insulin signaling, metabolism, inflammation	?	[30,70]
160b	↑	NAFLD	Human Biopsies	Insulin signaling	?	[30]
122	↓	NASH	HFD mice/Human Biopsies	Lipid and cholesterol metabolism	HMGCR, FAS, SREBP1/2, ACC	[66,71]
301a-3p	↑	Steatosis/NAFLD/NASH	Human Biopsies	?	?	[69]
375	↓	NAFLD/NASH/Cirrhosis	Human Biopsies	?	?	[69]
576-5p	↑	NAFLD	Human Biopsies	Insulin signaling, metabolic homeostasis, inflammation	mTOR signaling, ephrin B signaling, ROS production, RAC1	[70]
892a	↑	NAFLD	Human Biopsies	Kupffer cell activation ?	?	[70]
I137	↑	NAFLD	Human Biopsies	?	?	[70]
1282	↓	NAFLD	Human Biopsies	Insulin signaling, metabolism, inflammation	?	[70]
3663-5p	↑	NAFLD	Human Biopsies	Insulin signaling, metabolism, inflammation	?	[70]
3924	↑	NAFLD	Human Biopsies	Insulin signaling, metabolism, inflammation	?	[70]
